# Nonlinear polaritons in a monolayer semiconductor coupled to optical bound states in the continuum

**DOI:** 10.1038/s41377-020-0286-z

**Published:** 2020-04-09

**Authors:** Vasily Kravtsov, Ekaterina Khestanova, Fedor A. Benimetskiy, Tatiana Ivanova, Anton K. Samusev, Ivan S. Sinev, Dmitry Pidgayko, Alexey M. Mozharov, Ivan S. Mukhin, Maksim S. Lozhkin, Yuri V. Kapitonov, Andrey S. Brichkin, Vladimir D. Kulakovskii, Ivan A. Shelykh, Alexander I. Tartakovskii, Paul M. Walker, Maurice S. Skolnick, Dmitry N. Krizhanovskii, Ivan V. Iorsh

**Affiliations:** 1 ITMO University, Saint Petersburg, 197101 Russia; 2 St. Petersburg Academic University, Saint Petersburg, 194021 Russia; 3 Saint Petersburg State University, ul. Ulyanovskaya 1, Saint Petersburg, 198504 Russia; 4 Institute of Solid State Physics, RAS, Chernogolovka, 142432 Russia; 5 Science Institute, University of Iceland, Dunhagi 3, IS-107, Reykjavik, Iceland; 6 Department of Physics and Astronomy, University of Sheffield, Sheffield, S3 7RH UK

**Keywords:** Photonic crystals, Polaritons, Nonlinear optics

## Abstract

Optical bound states in the continuum (BICs) provide a way to engineer very narrow resonances in photonic crystals. The extended interaction time in these systems is particularly promising for the enhancement of nonlinear optical processes and the development of the next generation of active optical devices. However, the achievable interaction strength is limited by the purely photonic character of optical BICs. Here, we mix the optical BIC in a photonic crystal slab with excitons in the atomically thin semiconductor MoSe_2_ to form nonlinear exciton-polaritons with a Rabi splitting of 27 meV, exhibiting large interaction-induced spectral blueshifts. The asymptotic BIC-like suppression of polariton radiation into the far field toward the BIC wavevector, in combination with effective reduction of the excitonic disorder through motional narrowing, results in small polariton linewidths below 3 meV. Together with a strongly wavevector-dependent *Q*-factor, this provides for the enhancement and control of polariton–polariton interactions and the resulting nonlinear optical effects, paving the way toward tuneable BIC-based polaritonic devices for sensing, lasing, and nonlinear optics.

## Introduction

Optical bound states in the continuum (BICs), supported by photonic crystal structures of certain geometries, have received much attention recently as a novel approach to generating extremely spectrally narrow resonant responses^1,2^. Since BICs are uncoupled from the radiation continuum through symmetry protection^3^ or resonance trapping^4^, they can be robust to perturbations of photonic crystal geometric parameters^5^. This robustness enables a broad range of practical applications, including recently demonstrated spectral filtering^6^, chemical and biological sensing^7,8^, and lasing^4^.

Providing an efficient light-trapping mechanism, optical BICs are particularly attractive for enhancing nonlinear optical effects^9,10^, with recent theoretical proposals discussing enhanced bistability^11^ and Kerr-type focusing nonlinearity^12^. However, for the practical realization of these proposals, a significantly stronger material nonlinear susceptibility than that generally available in dielectric-based photonic crystals is needed.

An attractive approach to the enhancement of the effective nonlinearity is to use exciton-polaritons—hybrid quasiparticles that inherit both the coherent properties of the photonic modes and the interaction strength of the excitons^13–15^. Hybrid nanophotonic systems incorporating atomically thin transition metal dichalcogenides (TMDs) have emerged as a particularly promising platform owing to their ease of fabrication and the possibility of room-temperature operation^16–18^. In addition to conventional microcavity-based designs, TMD exciton-polaritons have been observed in plasmonic lattices^19^, photonic crystal slabs (PCSs)^20,21^, and other nanophotonic structures^22^.

Coupling TMD excitons to optical BICs in photonic crystals will not only boost the potentially achievable nonlinearities but also provide control on the resonant BIC properties through the excitonic fraction in the polariton, as has been proposed theoretically^23^.

Here, we experimentally demonstrate and investigate nonlinear polaritons formed via the strong coupling of excitons in monolayer (1 L) MoSe_2_ and optical BICs in a 1D PCS, with Rabi splitting of >27 meV and BIC-like radiation suppression in the surface-normal direction. Despite the large ∼9 meV inhomogeneous broadening of the MoSe_2_ excitonic line, we achieve a small polariton linewidth below 3 meV, corresponding to a very well resolved splitting-to-linewidth ratio of ∼9 and *Q*-factors up to 900. Using the strongly wavevector-dependent *Q*-factor of the photonic crystal dispersion, we show a controllable reduction in the polariton linewidth by a factor of 5–10 when approaching the BIC. The narrow polariton lines allow us to accurately measure the polariton–polariton interaction strength through power-dependent spectral blueshifts in the resonant reflectance experiment, corresponding to an exciton–exciton interaction strength of *g*_*X*_ ∼ 1.0 μeV μm^2^. This polariton nonlinearity is comparable with the values measured in III–V materials^24,25^ and significantly larger than those previously observed in TMD monolayer-based systems^22^, paving the way toward quantum applications of exciton-polaritons in atomically thin semiconductors.

**Fig. 1:**
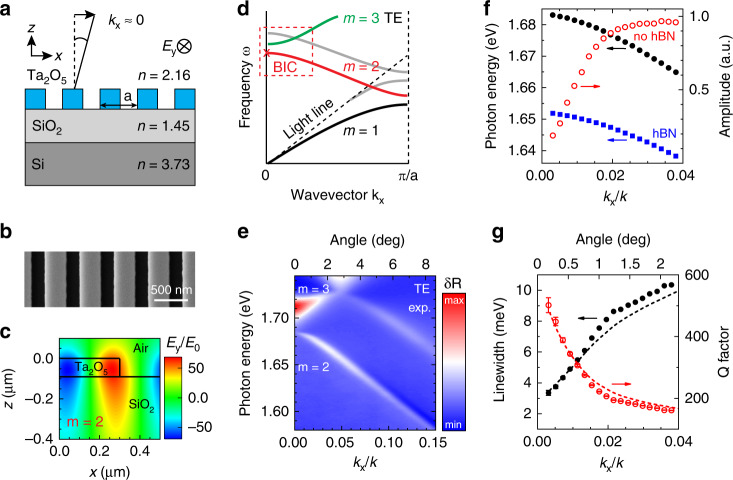
At-Γ optical BIC in a photonic crystal slab. **a** Schematic of a photonic crystal slab (PCS) sample, with Ta_2_O_5_ bars on a SiO_2_/Si substrate, illuminated with TE-polarized light near normal incidence. **b** SEM image of the PCS sample. **c** Calculated TE-mode electric field distribution at *k*_*x*_/*k* = 0.024. **d** Schematic of the photonic band structure for TE modes, with the at-Γ optical BIC position indicated with an *x*. **e** Experimental differential angle-resolved reflectance spectra showing one symmetric and two antisymmetric modes. **f** Wavevector-dependent peak position (black) and amplitude (red) extracted using Fano-like fits for the antisymmetric mode near the BIC location; blue squares show the peak positions shifted owing to a 9 nm hexagonal boron nitride (hBN) layer. **g** Extracted wavevector-dependent linewidth (black) and *Q*-factor (red), together with the corresponding simulation results (dashed lines) corrected for experimental resolution and scattering losses

In the experiment, we fabricate a 1D PCS sample consisting of 90 nm thick Ta_2_O_5_ bars on a SiO_2_/Si (1 μm/500 μm) substrate, as schematically shown in Fig. 1a, with a scanning electron microscopy image shown in Fig. 1b. The PCS geometry (see “Methods”) is designed for large refractive index modulation to open a photonic band gap and support an optical BIC close to the exciton resonance in monolayer MoSe_2_. As illustrated in the photonic band structure shown in Fig. 1d, the BIC is expected to form on the lower-energy *m* = 2 TE mode (red) at the *Γ* point in the crystal momentum space^23^, with characteristic confinement and antisymmetric spatial distribution of the optical field (c) with respect to the mirror symmetry plane of the PCS cell (see Supplementary Fig. S6).

We measure the PCS band structure via angle-resolved reflectance spectroscopy (see “Methods” and Supplementary Fig. S1). Figure 1e shows the experimental differential reflectance spectra for varying angle *θ*, where the signal from the un-patterned Ta_2_O_5_/SiO_2_/Si substrate is subtracted for clarity: *δR* (*θ*, *ω*) = *R*_PCS_ (*θ*, *ω*) − *R*_Sub_ (*θ*, *ω*). Three modes, a broad symmetric one (*m* = 3) and two narrower antisymmetric ones (*m* = 2), are clearly observed in the figure, in agreement with the theory (d, red dashed box). We fit the lower-energy antisymmetric mode peak in the reflectance spectra using a Fano-like line shape *F*(*ω*) ∝ (*qγ*/2 + *ω* − *ω*_0_)^2^/(*γ*^2^/4 + (*ω* − *ω*_0_)^2^) with resonance frequency *ω*_0_, linewidth *γ*, and asymmetry parameter *q*, which arises due to interference with the broad symmetric mode and an even broader Fabry–Perot response of the layered substrate (see Supplementary Fig. S2).

The extracted Fano fit parameters are plotted in Fig. 1f, g as functions of the ratio *k*_*x*_/*k* = sin*θ*, where *k*_*x*_ is the in-plane wavevector component, *k* is the free-space wavevector magnitude, and *θ* is the angle with respect to the surface normal. Toward the *Γ* point (*k*_*x*_ → 0), the reflectivity associated with the mode sharply decreases (f, red), while the spectral line narrows (g, black circles), resulting in a sharply increasing *Q*-factor (g, red open circles), defined as *Q* = *ω*_0_/γ. This behavior is a characteristic of an at-Γ optical BIC^26^, where the interference of optical waves outgoing in opposite directions leads to effective light trapping in the near field and vanishing far-field radiation. This is in contrast to the case studied recently^20,21^, where only radiating PCS modes with smaller and largely angle-independent *Q-*factors were considered for strong coupling to excitons in 2D semiconductors.

The theoretically predicted radiative *Q*-factor of the BIC diverges toward infinity at *k*_*x*_ = 0. In practice, the measured *Q*-factor is limited predominantly by nonradiative losses^5,26^. The two major contributions in our case are (1) intrinsic absorption in Ta_2_O_5_ and (2) surface-roughness-induced symmetry breaking and scattering^27^, limiting *Q* to ~10^3^. Additional resonance broadening comes from leaky losses in the Si due to near-field penetration through the SiO_2_ layer^27^ and finite sample size effects^3,28^.

We simulate the PCS dispersion and associated *Q*-factors with the Fourier modal method, taking into account these four loss mechanisms (see Supplementary Note 1 and Fig. S3). As shown in Fig. 1g (dashed lines), good agreement with the experiment can be achieved by considering the scattering losses through an additional imaginary part of the Ta_2_O_5_ refractive index δ*n* ∼ 0.002*i* (Fig. 1g, dashed lines).

**Fig. 2:**
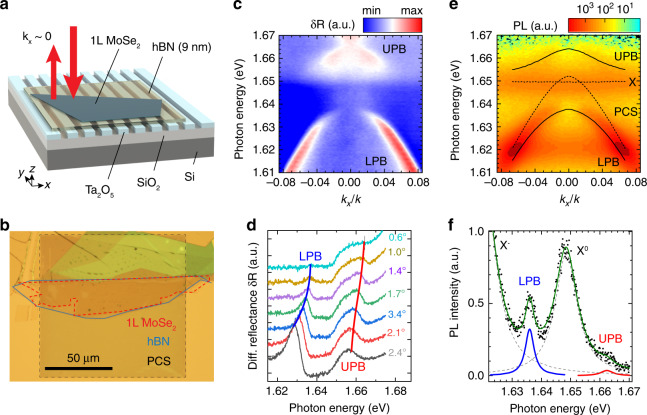
Strong coupling of excitons in 1 L MoSe_2_ and BIC. **a** Schematic of a hybrid 1 L MoSe_2_/hBN/PCS structure. **b** Optical microscope image of the fabricated sample. **c** Angle-resolved reflectance spectra of the hybrid sample, showing the upper and lower polariton branches due to strong coupling between the MoSe_2_ exciton and antisymmetric TE mode of the PCS. **d** Differential reflectance spectra for selected angles. **e** Angle-resolved PL spectra (TE-polarized), with the positions of the uncoupled PCS mode and MoSe_2_ exciton (dashed lines) and the resulting polaritons (solid lines) indicated. **f** Experimental PL spectrum for *k*_*x*_/*k* = 0.015 (dots) and the Lorentz fits. The dashed lines indicate fits for uncoupled exciton and trion peaks, and the blue and red lines indicate fits for the lower and upper polariton peaks, respectively

We then create polaritons by coupling the observed BIC to excitons in the monolayer MoSe_2_ in a vertically stacked structure consisting of 1 L MoSe_2_, multilayer hexagonal boron nitride (hBN), and a PCS, as illustrated in Fig. 2a. To maximize the *Q*-factor of the resulting polariton modes, we use large-area multilayer hBN and monolayer MoSe_2_ flakes of ∼100 μm in size, covering ∼200 periods of the PCS, as demonstrated in the optical microscope image in (b). The hBN spacer plays a threefold role: it avoids MoSe_2_ flakes “sagging” into the PCS grooves, reduces the influence of the Ta_2_O_5_ surface roughness, and provides tunability of the BIC frequency through the hBN thickness. In our case, a 9 nm thick hBN spacer shifts the PCS mode and spectral position of the BIC by ∼30 meV to bring it close to resonance with the neutral exciton in the 1 L MoSe_2_ at 7 K, with *ħω*_*X*_ = 1.65 eV (Fig. 1f, blue squares).

We study the polaritons experimentally via angle-resolved reflectivity and photoluminescence (PL) measurements at 7 K (see “Methods”), with the results of TE-polarized detection shown in Fig. 2c–f. In comparison with Fig. 1e, the lower-energy antisymmetric PCS mode observed in reflectivity (Fig. 2c, d) is now redshifted by ∼30 meV owing to the presence of hBN/MoSe_2_ and split into upper and lower polariton branches (UPB and LPB, respectively) owing to strong coupling with the neutral exciton in the 1 L MoSe_2_ centered at *ħω*_*X*_ = 1.65 eV (see also Supplementary Fig. S4). Both the LPB and UPB retain BIC-like behavior near the *Γ* point, exhibiting several distinctive properties.

First, at the *Γ* point, both the LPB and UPB are “dark,” as radiation into the far field becomes symmetry forbidden, effectively extending the interaction time for potential enhancement of the optical nonlinearities. Second, close to the *Γ* point, polaritons possess a negative effective mass and associated negative group velocity inherited from the PCS mode, providing a potential platform for studying TMD-based polariton self-focusing and soliton formation, similar to what has been discussed theoretically and studied experimentally in other polariton systems^14,29^. Third, the strong variation in the PCS mode linewidth in the vicinity of the BIC results in a wavevector-dependent *Q*-factor of both the LPB and UPB, enabling control of the polariton linewidth with the angle. These strongly modulated *Q*-factors, when combined with polariton–polariton interactions, can lead to novel phenomena such as the emergence of the so-called “weak lasing” state of matter^30^ or the spontaneous formation of superfluid polariton currents^31^.

Further details of the optical response are revealed by TE-polarized angle-resolved PL spectra (e), showing emission from both polariton branches and the uncoupled neutral exciton (X^0^). The latter is increasingly enhanced toward small wavevectors and exhibits a slight redshift of ∼1 meV, which we attribute to weak coupling to the higher frequency and broader *m* = 3 symmetric mode (see Supplementary Note 2 and Fig. S5). Charged exciton (trion, X^−^) emission is also observed at *ħω*_*T*_ = 1.62 meV independent of *k*_*x*_, implying a weak coupling.

**Fig. 3:**
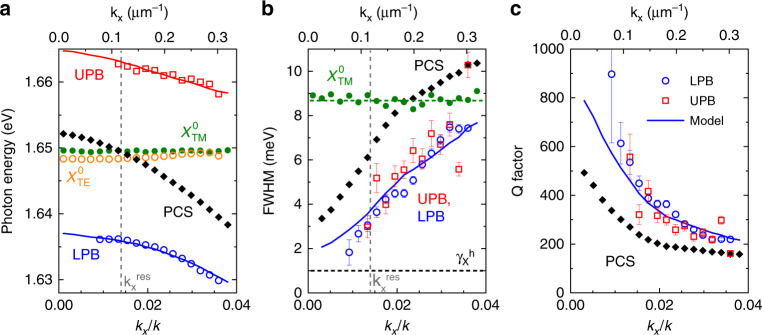
Motional narrowing for BIC-based exciton-polaritons. **a** Spectral peak position, **b** linewidth as the full width at half maximum (FWHM), and **c** corresponding *Q*-factor for the PCS mode (black diamonds), lower polariton branch (LPB, blue circles), and upper polariton branch (UPB, red squares), extracted from fits and compared with the coupled harmonic oscillator model. Also shown are the parameters of the MoSe_2_ neutral exciton in the TM polarization (**a**, **b** green circles), the peak position of the uncoupled exciton in the TE polarization (**a** orange circles), and the estimated homogeneous linewidth of the excitons $\gamma _{\mathrm{X}}^h$ (**b** black dashed line). The blue lines in **b** and **c** show the model calculations for the UPB FWHM and *Q*-factor

We analyze the wavevector-dependent behavior of the LPB, UPB, uncoupled neutral exciton, and trion by fitting the PL spectra for each *k*_*x*_ with four Lorentzian functions *L*_*i*_ (*ω*) ∝ ((*ω* − *ω*_*i*_)^2^ + (*γ*_*i*_/2)^2^)^−1^, as shown in Fig. 2f, and extracting the spectral position *ω*_*i*_ and linewidth *γ*_*i*_ as the full width at half maximum (FWHM) for each peak. The extracted parameters are plotted as functions of the in-plane wavevector in Fig. 3, with spectral positions (a) for the UPB (red symbols), LPB (blue symbols), and uncoupled neutral exciton (orange symbols), corresponding values of the FWHM (b), and calculated *Q*-factors (c). The parameters for the uncoupled excitons extracted from the TM-polarized PL are plotted as green dots.

We then fit the extracted spectral positions of the UPB (*ω*_+_) and LPB (*ω*_*−*_) with a coupled oscillator model^32^, using the spectral position and homogeneous linewidth for the uncoupled neutral exciton $\tilde \omega _{X} = {\omega}_{X} + i\gamma _{X}$ and for the PCS/hBN mode $\tilde \omega _{C}\left( {k_x} \right) = \delta \omega _{C} + \omega _{C}\left( {k_x} \right) + i\gamma _{C}\left( {k_x} \right)$:

$${\omega}_{\pm}=\operatorname{Re}\left[\frac{{\tilde{\omega}}_{C}+{{\tilde{\omega}}_{X}}}{2} \pm \frac{1}{2}{\sqrt{{{\hbar}^{2}}{{\Omega}_{R}^{2}}+({\tilde{\omega}}_{C}-{\tilde{\omega}}_{X})^{2}}}\right],$$

$$\hbar{\Omega_R}=2\sqrt{{\kappa}^{2}-\frac{({\gamma_C}-\gamma_{X})^2}{4}}.$$

Here, Ω_*R*_ is the Rabi splitting between the UPB and LPB, and the two fit parameters are the coupling strength *κ* and additional spectral shift *δω*_*C*_ of the PCS mode^20^ due to the presence of 1 L MoSe_2_. The fit curves for the spectral positions of the UPB and LPB are shown in Fig. 3a by the red and blue solid lines, respectively. The uncoupled PCS/hBN mode, indicated by the black squares, comes into resonance with the uncoupled neutral exciton at $k_x^{{\mathrm{res}}}/k \simeq \pm 0.014$, corresponding to an angle of ${\theta^{\mathrm{res}}} \simeq \pm 0.8^\circ$. From the fits in Fig. 3a, we extract a coupling strength of *κ* = 13.9 meV, which corresponds to a Rabi splitting of Ω_*R*_ = 27.4 meV and splitting-to-linewidth ratio of ∼9, exceeding the values recently reported for a WSe_2_/PCS system^20^ and theoretical estimates for strong coupling to an optical BIC^23^. Because Ω_*R*_ is larger than the sum of the exciton (∼9 meV) and PCS mode linewidth (∼3−11 meV), the hybrid MoSe_2_/hBN/PCS system is unambiguously in the strong coupling regime.

Quantitatively, the polariton linewidth *γ*_±_ is expected to vary between that of the exciton (*γ*_*X*_) and PCS modes (*γ*_*C*_) depending on the excitonic fraction in the polariton. However, our experimentally observed values of the polariton linewidth (Fig. 3b, open symbols) close to resonance $k_x = k_x^{{\mathrm{res}}}$ are significantly smaller than both *γ*_*X*_ (green) and *γ*_*C*_ (black). We attribute this to polariton motional narrowing, similar to the effects studied previously for quantum wells in microcavities^33–37^.

Here, the large polariton mode size (tens of μm) together with the large Rabi splitting lead to effective averaging over excitonic disorder^38^ in the 1 L MoSe_2_ over a broad (nm–μm) range of length scales. As a result, the excitonic contribution to the polariton FWHM close to resonance is given by only the homogeneous exciton linewidth $\gamma _{\mathrm{X}}^h$^39^, while away from resonance, where the polariton frequency overlaps with the exciton peak, it changes toward the inhomogeneous linewidth $\gamma _{\mathrm{X}}^{inh}$ due to an increasing interaction with disorder and associated scattering with higher momenta excitonic states as well as absorption^36,40^. We use a phenomenological model that accounts for homogeneous and inhomogeneous contributions to the polariton linewidth (see Supplementary Note 3). The model shows good agreement with the experimental data (Fig. 3b, blue curve) for a homogeneous linewidth of $\gamma _{\mathrm{X}}^h\sim 1$ meV (b, black dashed line), which is within the range of recently reported values^41–44^ for the low-temperature radiative decay rate of excitons in monolayer MoSe_2_.

As a result of excitonic and photonic disorder averaging, the *Q*-factors achieved for polaritons around the *Γ* point in our structure are ∼2 times higher than those for the bare PCS mode (Fig. 3c), reaching *Q* ∼ 900. These higher *Q*-factors offer the potential for further improvement of the polariton linewidth through the fabrication of macroscopic photonic crystal samples^3^ with improved surface quality and the use of large TMD flakes grown by chemical vapor deposition. In addition, the strongly *k*-dependent *Q*-factor of the PCS mode in the vicinity of the BIC enables precise control of the polariton linewidth and corresponding *Q*-factors via angle or temperature tuning (see Supplementary Note 4 and Figs. S7 and S8).

**Fig. 4:**
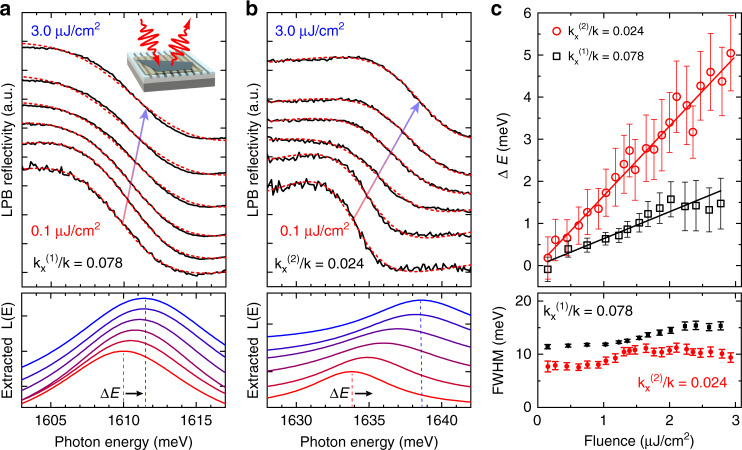
Nonlinear interaction of BIC-based polaritons. **a** Measured LPB reflectance spectra (solid black curves) under resonant illumination with laser pulses at $k_x^{\left( 1 \right)}/k = 0.078$ for fluence increasing from bottom to top, together with the corresponding Fano fits (red dashed curves). The inset illustrates the measurement geometry; the arrow indicates the power-dependent LPB frequency; and the bottom panel shows the corresponding Lorentzian curves based on the extracted frequency and linewidth. **b** Reflectance spectra, fits, and corresponding Lorentzian curves for $k_x^{\left( 2 \right)}/k = 0.024$. **c** Top panel: extracted LPB spectral blueshifts for laser excitation at $k_x^{\left( 1 \right)}/k = 0.078$ (black squares) and $k_x^{\left( 2 \right)}/k = 0.024$ (red circles) as functions of the incident fluence, together with the corresponding linear fits (black and red lines). Bottom panel: corresponding extracted linewidth (FWHM) as a function of the incident fluence

Mixing photonic modes with excitons in our hybrid MoSe_2_/hBN/PCS structures leads to a dramatic enhancement of the associated optical nonlinearities. We probe the underlying polariton–polariton interaction due to the excitonic contribution by measuring the pump-dependent frequency shifts of the polariton peaks in the reflectivity spectra. The polariton modes are excited resonantly in both the frequency and wavevector domains by ∼130 fs laser pulses (Fig. 4a, inset), with the incident fluence varying from 0.1 μJ/cm^2^ to 3.0 μJ/cm^2^ (see “Methods”).

Figures 4a, b show the measured pump-dependent reflectivity spectra of the LPB resonance (solid black curves) for selected values of the incident fluence, increasing from bottom to top, and for two different *x*-components of the wavevector: $k_x^{\left( 1 \right)}/k = 0.078$ (a) and $k_x^{\left( 2 \right)}/k = 0.024$ (b). Due to the exciton–exciton interaction, which increases with the density of the created quasiparticles^45^, the lower-energy polariton resonance in the reflectivity spectra is continuously shifted with the fluence toward higher energies, as clearly seen from the Lorentzian curves *L*(*E*) based on the resonance frequency and linewidth extracted from the fits (Fig. 4a, b, bottom panels). We observe larger blueshifts (b) for wavevectors closer to the anticrossing condition $k_x^{{\mathrm{res}}}/k = 0.014$, as expected for stronger polariton–polariton interactions associated with the increasing exciton fraction in the polariton.

The top panel in Fig. 4c shows the blueshift values for $k_x^{\left( 1 \right)}/k = 0.078$ (black squares) and $k_x^{\left( 2 \right)}/k = 0.024$ (red circles) extracted from the Fano line shape fitting (a, b, red dashed curves) for varying fluence, together with the corresponding linear fits (black and red lines). Calculating the polariton density *n*_*P*_ for each fluence (see Supplementary Note 5), we obtain the polariton–polariton interaction strength *g*_*P*_ = *dE*_*P*_/*dn*_*P*_ of $g_P( {k_x^{\left( 1 \right)}} ) \sim 0.04$ μeV μm^2^ and $g_P( {k_x^{\left( 2 \right)}} ) \sim 0.16$ μeV μm^2^. Furthermore, from the $g_P\left( {k_x} \right) \propto g_X\left| {X\left( {k_x} \right)} \right|^4$ dependence on the Hopfield coefficient *X*(*k*_*x*_), which describes the exciton fraction in the polariton, we estimate the exciton–exciton interaction strength in our measurement to be *g*_*X*_ = 1.0 ± 0.4 μeV μm^2^. This value is on the same order as the theoretical estimate *g*_*X*_ ~ 1.6 μeV μm^2^, as well as the value *g*_*X*_ ~ 1.4 μeV μm^2^ we extract from a direct measurement of the pump-dependent excitonic blueshifts in TM polarization (see Supplementary Note 6 and Fig. S9).

While it is difficult to directly compare our *g*_*X*_ values with those observed in III–V materials due to the few orders of magnitude difference in the reported numbers, our interaction strength is not much lower than the values of *g*_*X*_ ≃ 10 μeV μm^2^ recently extracted from careful measurements in GaAs-based polaritonic systems^25^. On the other hand, our nonlinearities are considerably larger than those reported previously for WS_2_-based polaritons^22^, where the estimation of the exciton–exciton interaction strength could possibly be uncertain due to efficient local heating at 300 K and the use of high excitation densities leading to higher-order interaction effects and associated redshifts.

The exciton densities in our experiment (≤10^12^ cm^−2^) are far below the Mott transition density (~10^14^ cm^−2^), and the observed polariton nonlinearity is mostly due to the exciton–exciton interaction, with phase space filling effects likely playing only a minor role. As seen from the fluence-dependent linewidth plots in the bottom panel of Fig. 4c, the increased interaction at higher densities also leads to a faster polariton decay, manifested as power-dependent broadening. In addition, we note that the observed nonlinearities are fast at least on a 100 fs scale, providing future opportunities for developing polariton-based ultrafast modulators and switches.

In summary, we present the first experimental demonstration and investigation of optical BIC-based polaritonic excitations. The formation of BIC-like polaritons in a hybrid system of a monolayer semiconductor interfaced with a PCS, with suppressed radiation into the far field and line narrowing due to effective disorder averaging, extends the polariton–polariton interaction time, which enhances the nonlinear optical response. In the future, these “dark” states can be accessed through near fields using guided modes excited by grating coupling or by nonlinear frequency conversion. With the strength of the underlying exciton–exciton interaction *g*_*X*_ ~ 1.0 μeV μm^2^, our polaritons exhibit strong exciton-fraction-dependent optical nonlinearities that are fast on a 100 fs time scale. In addition, the planar geometry of our structure allows straightforward fabrication of the electrical contacts for electrostatic control of the polaritons and associated interactions, while the use of atomically thin semiconductors in principle allows room-temperature operation. Thus, the formation of BIC-based polaritons can enable not only significantly enhanced but also controllable and fast nonlinear optical responses in photonic crystal systems due to the strong excitonic interaction in monolayer semiconductors and can open a new way to develop active and nonlinear all-optical on-chip devices.

## Methods

### Sample fabrication

Ta_2_O_5_ layers of 90 nm thickness were deposited on commercial SiO_2_/Si substrates via e-beam assisted ion-beam sputtering. PCSs were fabricated by patterning the Ta_2_O_5_ layers via a combination of electron-beam lithography and plasma etching to yield the following geometric parameters: pitch *p* = 500 nm, groove width *w* = 220 nm, and depth *d* = 90 nm, as characterized by scanning electron and atomic force microscopy measurements. Large-area high-quality flakes of multilayer hBN and monolayer MoSe_2_ were mechanically exfoliated from commercial bulk crystals (HQ Graphene) and stacked vertically onto the photonic crystal sample surface via dry transfer to form a hybrid 1 L MoSe_2_/hBN/PCS structure.

### Optical measurements

Angle-resolved reflectance spectroscopy was performed in a back-focal-plane setup with a slit spectrometer coupled to a liquid-nitrogen-cooled imaging CCD camera (Princeton Instruments SP2500+PyLoN), using white light from a halogen lamp for illumination (see Supplementary Fig. S1). For pump-dependent reflectivity measurements, the sample was excited by 130 fs pulses from a wavelength-tuneable Ti:sapphire oscillator (Spectra-Physics, Tsunami, 80 MHz repetition rate) with wavevector control via laser beam positioning within the back focal plane of the objective. A single-slit optical chopper with a duty cycle of 0.001 was used in the laser beam to avoid sample heating. Angle-resolved PL measurements were performed in the same setup with off-resonant excitation by monochromatic light from a HeNe laser with a wavelength λ_*exc*_ = 632.8 nm. The sample was mounted in an ultra-low-vibration closed-cycle helium cryostat (Advanced Research Systems) and maintained at a controllable temperature in the range of 7−300 K. The cryostat was mounted onto a precise xyz stage for sample positioning. Spatial filtering in the detection channel was used to selectively measure signals from the 1 L MoSe_2_/hBN/PCS sample area.

## Supplementary information

Supplementary Information

## Data Availability

The data that support the plots within this paper and other findings of this study are available from the corresponding authors upon reasonable request.
