# Supplementary material for: Nonlinear polaritons in a monolayer semiconductor coupled to optical bound states in the continuum
Source: Light Sci Appl. 2020 Apr 9;9:56. doi: 10.1038/s41377-020-0286-z (PMC7145813; doi:10.1038/s41377-020-0286-z)
Supplement: Supplementary file 1 — Supplementary Information [file 41377_2020_286_MOESM1_ESM.pdf]

Supplementary Information for

Nonlinear polaritons in a monolayer semiconductor coupled to optical bound states in the continuum

Vasily Kravtsov,[1,*] Ekaterina Khestanova,[1,†] Fedor A. Benimetskiy,[1] Tatiana Ivanova,[1] Anton K. Samusev,[1] Ivan S. Sinev,[1] Dmitry Pidgayko,[1] Alexey M. Mozharov,[2] Ivan S. Mukhin,[1,2] Maksim S. Lozhkin,[3] Yuri V. Kapitonov,[3] Andrey S. Brichkin,[4] Vladimir D. Kulakovskii,[4] Ivan A. Shelykh,[1,5] Alexander I. Tartakovskii,[6] Paul M. Walker,[6] Maurice S. Skolnick,[1,6] Dmitry N. Krizhanovskii,[6] and Ivan V. Iorsh[1,‡]

[1]*ITMO University, Saint Petersburg 197101, Russia*

[2]*St. Petersburg Academic University, Saint Petersburg 194021, Russia*

[3]*Saint Petersburg State University, ul. Ulyanovskaya 1, Saint Petersburg 198504, Russia*

[4]*Institute of Solid State Physics, RAS, Chernogolovka 142432, Russia*

[5]*Science Institute, University of Iceland, Dunhagi 3, IS-107, Reykjavik, Iceland*

[6]*Department of Physics and Astronomy, University of Sheffield, Sheffield S3 7RH, UK*

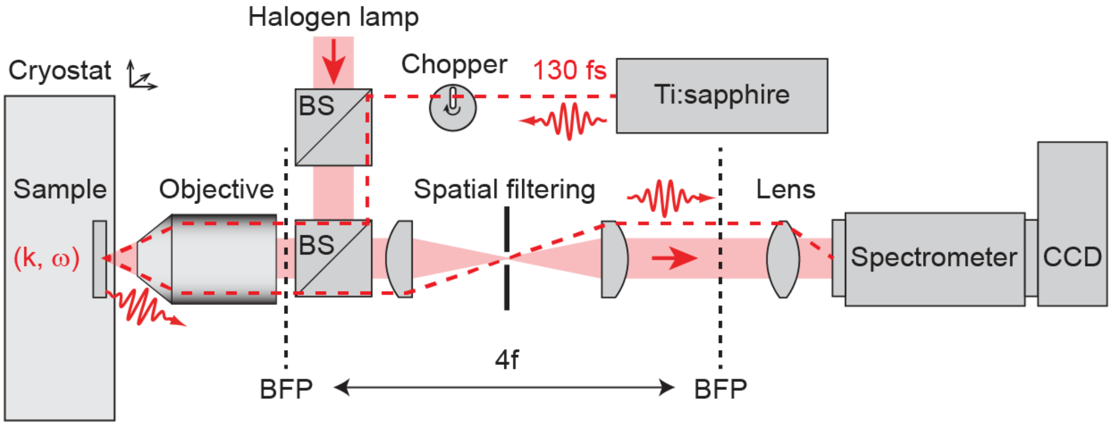

FIG. S1. Schematic of experimental setup for angle-resolved reflectivity measurements. A halogen lamp is used for measurements in the linear optical regime, and a tunable Ti:sapphire oscillator with 130 fs pulses for nonlinear experiments. A single-slit optical chopper with 0.001 duty cycle is used to reduce sample heating. Angle-resolved data are collected from the back focal plane (BFP) of a microscope objective with subsequent filtering in real space (in a 4f configuration) to eliminate unwanted background signal. Samples are mounted in a closed-cycle He-free cryostat with micrometric positioning along 3 spatial axes. Resonant laser excitation is achieved by focusing laser pulses in BFP with a corresponding ~ 200 µm focal spot and tuning Ti:sapphire oscillator into resonance with a polariton mode. All signals are detected with a slit spectrometer ($f = 500$ mm, 600 g/mm) and liquid nitrogen cooled CCD camera. BS: beamsplitter.

SUPPLEMENTARY NOTE 1. BIC Q-FACTOR SIMULATIONS

With the diverging to infinity radiative Q-factors of optical BICs in photonic crystal slabs (PCSs), their total Q-factors are limited by nonradiative terms, including material absorption, surface roughness induced scattering, leakage through the oxide layer into the Si substrate, and losses due to the finite sample size. The effects of material absorption and leakage into the Si substrate on the Q-factor can be directly estimated from simulations. Losses related to surface roughness induced scattering can be taken into account phenomenologically via an additional imaginary part of the Ta_2O_5 refractive index.

The effect of the finite sample size can be described as follows. Resonant modes in a finite structure are characterized by discrete wavenumbers quantized with an inverse length of the structure, rather than by a continuous in-plane wavevector. For specific geometries,

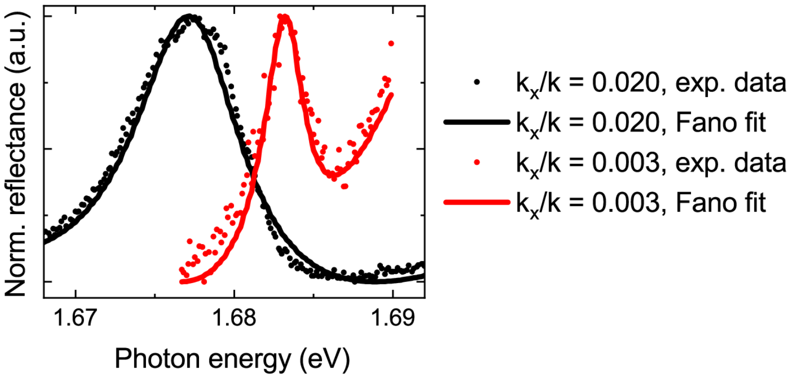

FIG. S2. Normalized differential reflectance spectra from a PCS sample for two selected wavevectors: $k_x/k = 0.020$ (black) and $k_x/k = 0.003$ (red). Experimental data are indicated with dots, and lines represent Fano-like fits. For different wavevectors, Fano parameter varies, resulting in different peak shapes. The shoulder on the blue side is due to the higher energy symmetric mode of the photonic crystal.

the quality factor is affected by several factors, including the shape of the terminating boundary and the dispersion of propagating modes. For example, the terminating boundary can be engineered to reduce the coupling to the propagating modes of the unpatterned structure [1]. The dispersion of the modes can also crucially modify the dependence of the Q-factor on the number of periods [2]. Specifically, for accidental bound states in the continuum occurring within the Brillouin zone, where the group velocity is finite, the quality factor grows linearly with the number of periods, yielding low quality factors even for large structures. In contrast, symmetry protected BICs occurring at the band edges, where the dispersion of modes is parabolic, are characterized by the quadratic dependence of the Q-factor on the number of periods, and thus low radiative losses can be achieved even in moderate-sized structures [3]. It should also be stressed that a finite detection spot size has a similar effect on the Q-factor. In our experiment, the detection spot size (D) was matched to the size of the periodic nanostructure, and therefore we can estimate the effect of the finite lateral size via an additional broadening in the Fourier domain $\Delta k \sim 1/D$.

To estimate Q-factors achievable in our experimental system, we perform simulations of its optical response using the Fourier modal method (FMM) [4]. We first simulate a patterned $Ta_2O_5/SiO_2/Si$ structure with the experimental parameters (Ta_2O_5 thickness of 90 nm, SiO_2 thickness of 1 µm, pitch p = 500 nm, groove width w = 220 nm, and depth

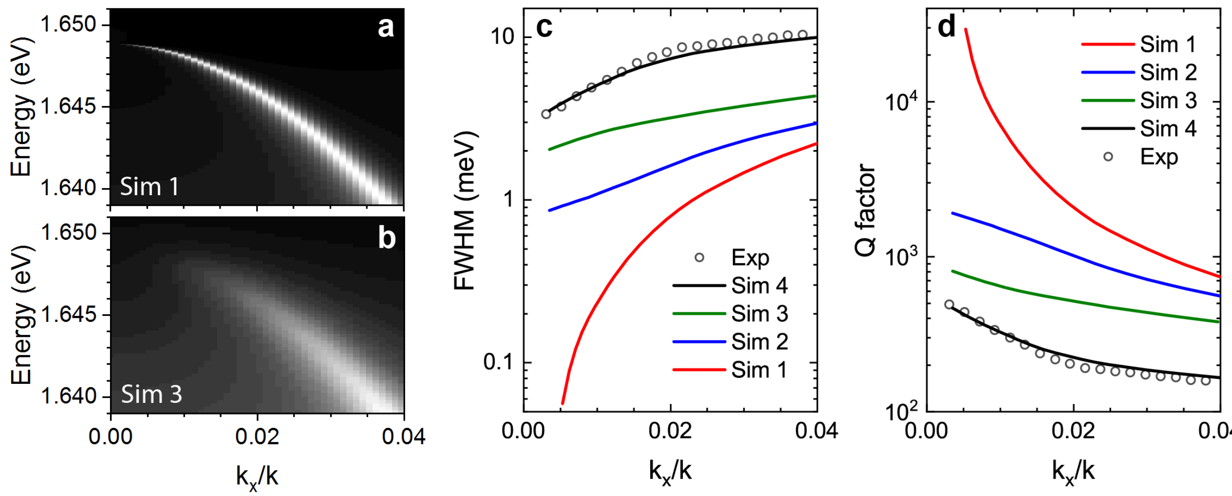

FIG. S3. Influence of losses on the $m = 2$ lower-energy antisymmetric mode in PCS: simulated reflectivity as a function of photon energy and x-component of wavevector without losses in Ta_2O_5 (a) and corresponding simulation result when taking into account intrinsic losses in Ta_2O_5 together with additional scattering losses $\delta n = 0.002i$. Linewidth (c) and quality factor (d) of the $m = 2$ lower-energy antisymmetric mode obtained in experiment (Exp, circles) and in simulations (Sim 1-4, lines). Sim 1 (red): no losses (except leakage into Si), Sim 2 (blue): only intrinsic losses in Ta_2O_5 are considered, Sim 3 (green): scattering-related losses are added, Sim 4 (black): intrinsic losses, scattering-related losses, and line broadening due to the finite sample size are taken into account.

$d = 90$ nm), but ignoring losses in Ta_2O_5 (setting the imaginary part of its refractive index to zero).

The resulting dispersion of the lower-energy $m = 2$ antisymmetric mode is shown as angle-resolved reflectivity spectra in Fig. S3a and labeled as Sim 1. Fitting the reflectivity spectra for different wavevectors with Fano-like shape as discussed in the main text, we obtain the wavevector dependence of the mode linewidth plotted in (c) with a red curve. The corresponding Q-factor of the mode is plotted in (d) as a red curve and reveals a sharp increase towards the Γ point, with Q-factors reaching $Q \sim 3 \times 10^4$ at $k_x/k = 0.005$ and growing further towards $Q \sim 10^5$. Note that these simulation results already incorporate the losses due to leakage into the Si substrate. Therefore, even higher Q-factors can be potentially achieved if leakage losses are further reduced through the use of, for example, thicker SiO_2 layers or fully suspended photonic crystal slabs.

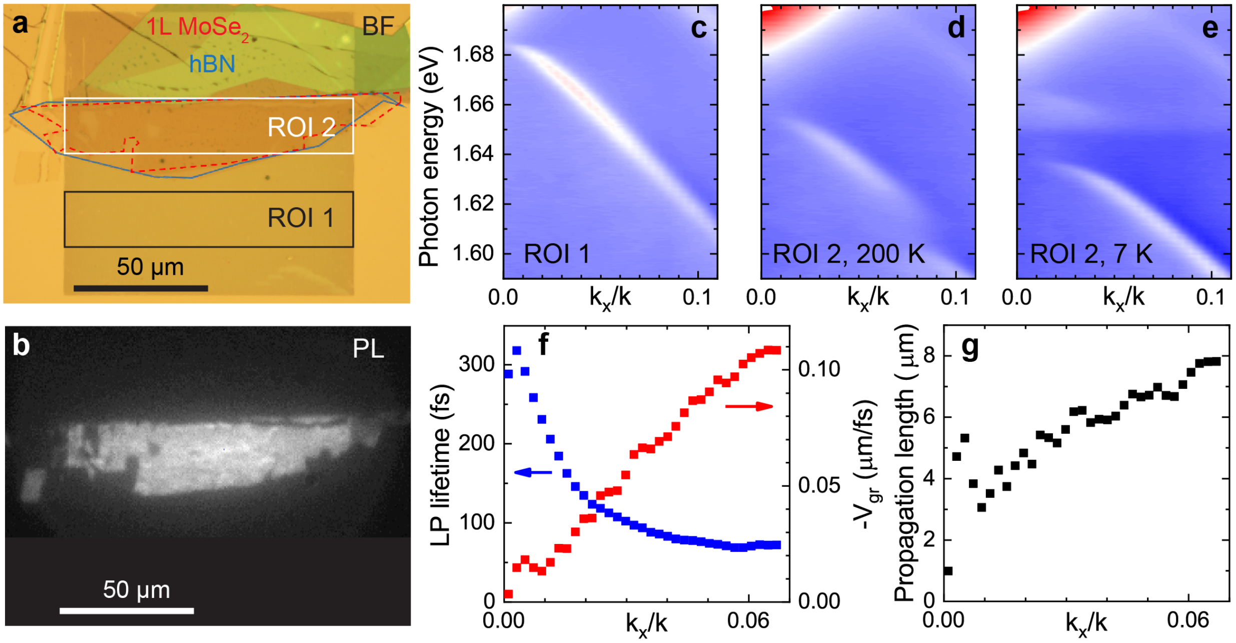

FIG. S4. Spatial filtering in the experiment. (a) Bright field (BF) microscope image of the sample, with 2 different regions of interest (ROI) for spatial filtering indicated with black (ROI 1) and white (ROI 2) rectangles. (b) Photoluminescence (PL) image of the sample taken in real space. (c) Reflectivity spectra taken in the momentum space for the bare PCS region (ROI 1). (d) Reflectivity spectra from ROI 2 taken at 200 K. (e) Reflectivity spectra from ROI 2 taken at 7 K. (f) Lifetime (blue) and group velocity (red) extracted from the measured lower polariton branch dispersion. (g) Propagation length extracted for the lower polariton branch.

In contrast, when taking into account the absorption losses in Ta_2O_5, the Q-factors are limited by $Q \sim 2 \times 10^3$, as indicated in (c) and (d) with a blue curve (labeled as Sim 2). To describe our experimental data, we account for additional scattering losses due to surface roughness via an extra imaginary term in the refractive index of Ta_2O_5, $\delta n \sim 0.002i$ (Sim 3), with linewidth and Q-factor shown in (c) and (d) with green curves, and dispersion shown in (b). Then, a convolution of the resulting lineshape with a Gaussian function that accounts for the finite sample size of $\sim$ 100 μm yields linewidth and corresponding Q-factor as shown with a black curve in (c) and (d). Experimental values (open black circles) are also plotted for reference. Overall, the absorption in Ta_2O_5 is seen to be the major limiting factor for the BIC linewidth in this case.

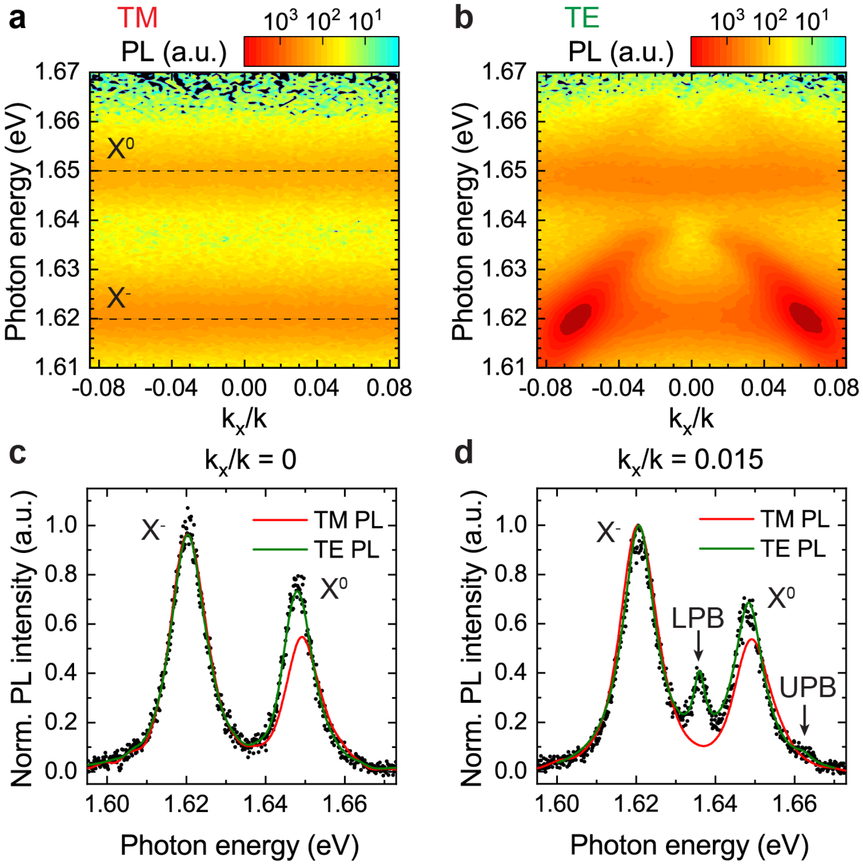

FIG. S5. Angle resolved PL spectra detected in TM (a) and TE (b) polarization. TM spectra show angle-independent neutral exciton (X^0) and trion (X^-) emission. TE spectra (b) in addition show upper and lower polariton branches. PL spectra for surface-normal emission (c) normalized to the trion peak intensity show slightly enhanced and redshifted neutral exciton emission in the TE polarization (green) as compared to TM polarization (red), due to weak coupling to the symmetric mode of the PCS. PL spectra for a small non-zero in-plane wave-vector component $k_x/k = 0.015$ (d, green) in addition show lower and upper polariton emission peaks (LPB, UPB), which are absent in the corresponding TM polarized PL spectrum (red).

SUPPLEMENTARY NOTE 2. MEASUREMENTS IN TE VS. TM POLARIZATION

In the TM polarization (E-field perpendicular to the grooves), the $MoSe_2$ exciton is far detuned from the photonic crystal mode. As shown in Fig. S5a, PL spectra detected in the TM polarization reveal only wavevector-independent uncoupled neutral exciton (X_0) and trion (X^-) emission. In the TE polarization, in addition to the upper and lower polariton

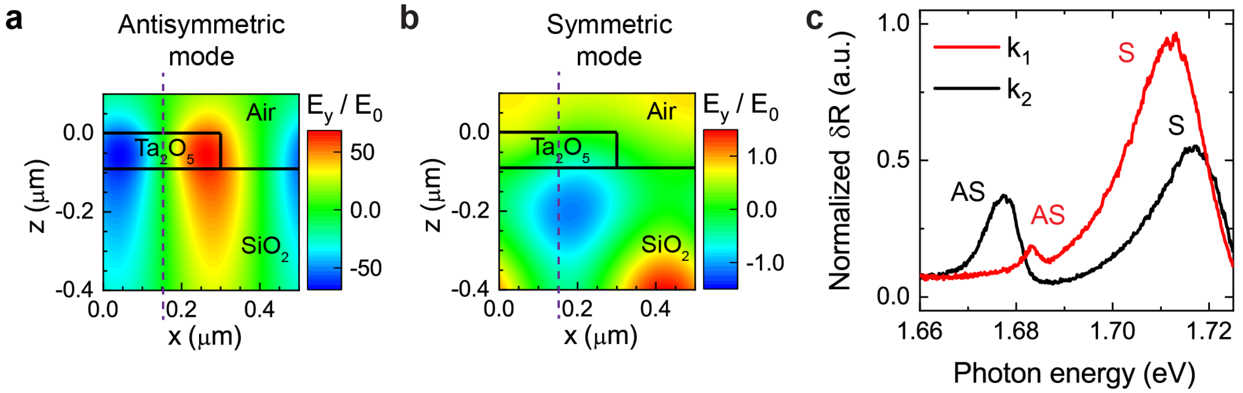

FIG. S6. Symmetric (S) and antisymmetric (AS) modes in PCS: spatial distribution of the y-component of electric field for AS (a) and S (b). The mirror symmetry plane of the PCS cell is indicated with dashed lines. Normalized differential reflectance spectra for two different wavevectors ($k_1 = 0.003k$, red, and $k_2 = 0.02k$, black), with distinct S and AS peaks (c).

branches as well as the trion peak, our experimental PL spectra (b) show emission close to the frequency of the uncoupled neutral exciton in 1L $MoSe_2$ with a slight redshift of ~ 1 meV and enhancement as compared to the spectra measured in the TM polarization (c).

This behavior is due to coupling to the $m = 3$ symmetric PCS mode lying at higher energies. While the strongly coupled to the exciton antisymmetric mode exhibits a tightly confined and enhanced electric field distribution (Fig. S6a), the symmetric mode is less confined (Fig. S6b), resulting in a weaker coupling, which, together with a larger detuning from the exciton resonance, leads to only a small "Lamb" redshift and slight enhancement of the exciton emission. The corresponding narrow antisymmetric (AS) and broader symmetric (S) peaks observed in the PCS reflectivity spectra are shown in (Fig. S6c) for two different wavevectors.

Due to the large detuning between the $MoSe_2$ exciton and PCS mode in the TM polarization, we can use the corresponding PL spectra to describe the original uncoupled $MoSe_2$ excitonic response. As shown in Fig. S5d, this provides a reference PL spectrum (red), which can be directly compared to that measured for small in-plane wavevectors in the TE polarization (green). The two additional peaks observed in TE-polarized PL are assigned to the lower and upper polariton emission, which provides compelling evidence for the strong coupling regime between $MoSe_2$ excitons and the antisymmetric PCS mode in our structure.

SUPPLEMENTARY NOTE 3. POLARITON LINEWIDTH MODEL

Since the Rabi splitting well exceeds the *inhomogeneous* exciton linewidth in our case, the polariton linewidth close to the anticrossing point ($\omega_{\mathrm{C}}(k_x^0) = \omega_{\mathrm{X}}$) will be defined through only the *homogeneous* contribution $\gamma_{\mathrm{X}}^{\mathrm{h}}$ to the exciton linewidth [5]. We describe the experimentally obtained polariton linewidth with a phenomenological model taking into account both homogeneous and inhomogeneous exciton linewidth, $\gamma_{\mathrm{X}}^{\mathrm{h}}$ and $\gamma_{\mathrm{X}}^{\mathrm{inh}}$. The effect of motional narrowing [6] results in the dependence of the polariton linewidth on the effective mass [7], which is incorporated in our model as

$$\gamma_{\pm} = |C_{\pm}|^2\gamma_{\mathrm{C}} + |X_{\pm}|^2\gamma_{\mathrm{X}}^{\mathrm{h}} + |X_{\pm}|^2\frac{M_{\pm}}{m_{\mathrm{x}}}\gamma_{\mathrm{X}}^{\mathrm{inh}}, \tag{1}$$

where $M_{\pm}$, m_{X} are effective masses of the polaritons and exciton, respectively, and $C_{\pm}$, $X_{\pm}$ are the Hopfield coefficients:

$$|C_{\pm}|^2 = \frac{\hbar^2\mathit{\Omega}_R^2}{|\hbar^2\mathit{\Omega}_R^2 + (\omega_{\pm} - \omega_{\mathrm{C}})^2|}, \quad |X_{\pm}|^2 = 1 - |C_{\pm}|^2. \tag{2}$$

An agreement between the model and experimental LPB linewidth is reached for $\gamma_{\mathrm{X}}^{\mathrm{h}} \sim$ 1.0 meV, which is close to recently reported values [8–10] for the low-temperature radiative decay rate of excitons in monolayer $MoSe_2$ of $\sim 1 - 2$ meV, implying that the homogeneous non-radiative contribution to $MoSe_2$ exciton linewidth at 7 K is small. Assuming a Voigt profile of the exciton line at 7 K, we estimate the inhomogeneous linewidth of $\gamma_{\mathrm{X}}^{\mathrm{inh}} \sim 8.4$ meV. The total homogeneous linewidth $\gamma_{\mathrm{X}}^{\mathrm{h}} = \gamma_{\mathrm{X}}^{\mathrm{nrad}} + \gamma_{\mathrm{X}}^{\mathrm{rad}}$ is then used to fit our experimentally measured dispersion with the coupled oscillator model at each temperature.

We note additionally that the experimentally observed polariton linewidth and Q-factor for small in-plane wavevectors are affected by the finite size of the overlap region between the $MoSe_2$ flake and PCS, similarly to the case of the bare PCS described in Supplementary Note 2. Namely, since the polariton dispersion near the $\mathit{\Gamma}$ point is also parabolic, the Q-factor scales quadratically with the number of PCS periods covered by $MoSe_2$. This yields high Q-factors even for moderately sized polariton systems. We note that the observed in Fig. 3b in the main text slight deviation between the model and experimentally extracted values close to the $\mathit{\Gamma}$ point might be due to this finite size effect.

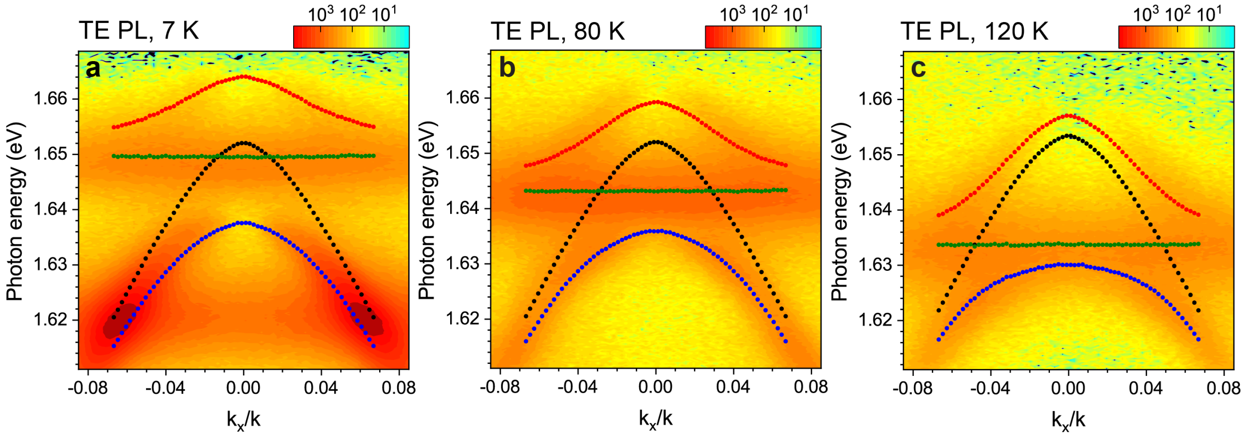

FIG. S7. Temperature tuning of the polariton dispersion, as observed from selected PL spectra in TE polarization at 7 K (a), 80 K (b), and 120 K (c). Black: PCS mode; green: neutral exciton; red: upper polariton branch; blue: lower polariton branch. Spectral peak positions are extracted at each angle by fitting PL spectra with Lorentzian functions.

SUPPLEMENTARY NOTE 4. VARIABLE TEMPERATURE MEASUREMENTS

With increasing temperature, the neutral $MoSe_2$ exciton shifts towards lower energies, effectively tuning the polariton dispersion. Fig. S7 shows experimental angle-resolved PL spectra for 3 selected temperatures of 7 K (a), 80 K (b), and 120 K (c), with the uncoupled PCS mode dispersion (black), uncoupled exciton (green), and upper and lower polariton branches (red and blue, respectively), as obtained by fitting the PL data with the coupled oscillator model. The temperature dependence of the polariton dispersion is also observed in reflectivity spectra (Fig. S8a-d). We extract a temperature-dependent coupling constant $g(T)$ (e, blue triangles) and compare it with the exciton linewidth (black squares). The clear signatures of strong coupling persist up to at least 150 K where exciton broadening starts to blur the observed anticrossing behavior.

SUPPLEMENTARY NOTE 5. POLARITON NONLINEARITY

The nonlinear polaritonic shift $\mathit{\Delta} E_{pol}$ can be estimated as [11]

$$\mathit{\Delta} E_{pol} \approx g_{\mathrm{X}} |X|^4 \int d^2\mathbf{r} |\psi(\mathbf{r})|^4 \left[\int d^2\mathbf{r} |\psi(\mathbf{r})|^2 \right]^{-1}, \tag{3}$$

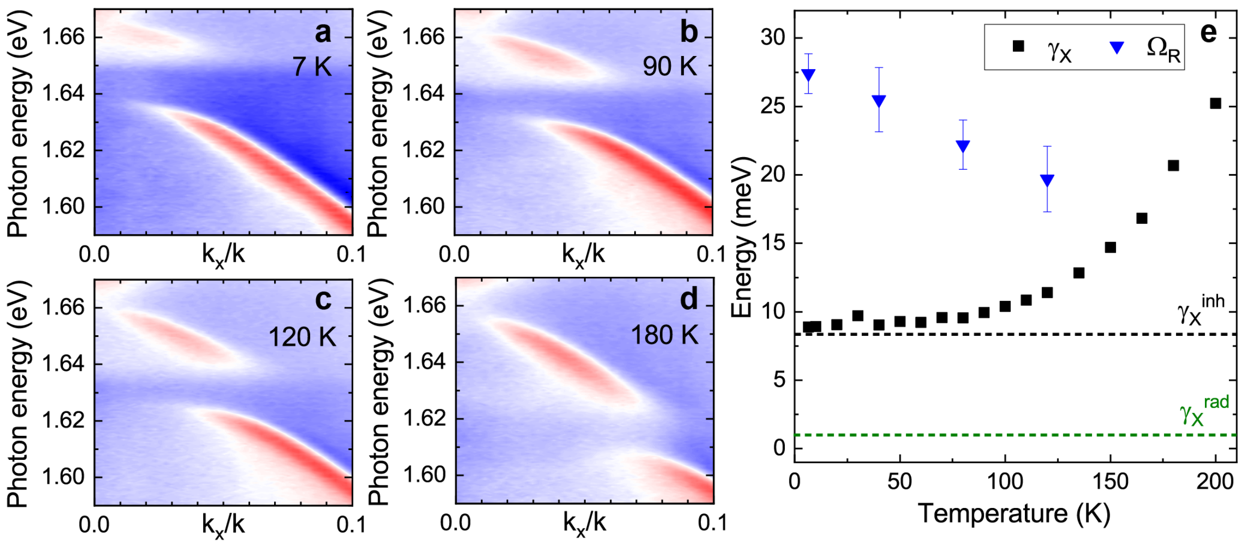

FIG. S8. Temperature dependence of $MoSe_2$/hBN/PCS optical response: (a-c) Selected angle-resolved reflectance spectra measured at 7 K, 90 K, 120 K, and 180 K. (e) Extracted Rabi splitting (blue triangles) and total exciton linewidth (black squares) for temperature in a range of $7-200$ K. Estimated radiative exciton linewidth γ_X^{rad} and inhomogeneous linewidth due to disorder γ_X^{inh} are indicated with green and black dashed lines, respectively.

where g_X is the exciton–exciton interaction constant, X is the exciton Hopfield coefficient for the corresponding polariton mode, and $\psi(r)$ is the polariton wavefunction. For the pulsed excitation case, ψ is time-dependent; for the nonlinear shift modelling, the wavefunction is evaluated at the moment of time when it acquires the largest value.

For a pulsed pump with spectral width γ_{pump} and polaritonic mode with radiative decay rate γ_0, the polaritonic wavefunction can be estimated (in the rectangular pulse shape approximation) as

$$\psi((r)) \approx \psi_{sat}(\mathbf{r})(1 - \exp[-\gamma_0/\gamma_{pump}]), \tag{4}$$

where ψ_{sat} is the saturated wavefunction, which corresponds to the case of continuous wave excitation.

The saturated wavefunction can be estimated as

$$\psi_{sat}(x) = \sqrt{\frac{W_{max}}{c\hbar\omega_p}}\sqrt{\int \mathcal{E}^2(x,z)dz} = \sqrt{\frac{W_{max}}{c\hbar\omega_p}}\phi(x), \tag{5}$$

where W_{max} is the peak power density, ω_p is the polariton frequency, $\mathcal{E}$ is the dimensionless field distribution of the eigenmode when it is excited by a plane wave of the unity amplitude

and frequency ω_p, and $\phi(x)$ is introduced to shorten the notation. $\mathcal{E}$ is calculated numerically using the Fourier modal method (FMM). It should be noted though that the general result of the temporal coupled mode theory [12] states that $\mathcal{E}$ is proportional to ${\gamma_0}^{-1/2}$.

The spectral shift due to polariton–polariton interaction is then given by

$$\mathit{\Delta} E_{pol} = g_{\mathrm{X}}|X|^4 \frac{W_{max}(1-\exp[-\gamma_0/\gamma_{pump}])^2}{c\hbar\omega_p} \frac{\int_0^D dx|\phi(x)|^4}{\int_0^D dx|\phi(x)|^2}, \tag{6}$$

where D is the period of the structure. We note that the last factor in the equation can lead to an effective enhancement of the nonlinear spectral shift for the case of well localized optical field vs. uniform field distribution. The nonlinear spectral shift is associated with the effective polariton density $\langle n_{pol}\rangle$:

$$\mathit{\Delta} E_{pol} = g_{\mathrm{X}}|X|^4 \langle n_{pol}\rangle. \tag{7}$$

Finally, the effective polariton density can be calculated as

$$\langle n_{pol}\rangle = \frac{W_{max}(1-\exp[-\gamma_0/\gamma_{pump}])^2}{c\hbar\omega_p} \frac{\int_0^D dx|\phi(x)|^4}{\int_0^D dx|\phi(x)|^2}. \tag{8}$$

The upper bound for the polariton density for the maximum fluence of 3 μJ cm^{-2} can be estimated as $n_{pol}^{max} \sim 10^{12}$ cm^{-2}. Thus, we are quite far from the Mott transition densities $a_b^{-2} \approx 10^{14}$ cm^{-2}, and phase space filling effects likely play only a minor role.

The values of $|X|^2$ and γ_0/γ_{pump} can be calculated for each wavevector. For example, for the two wavevectors corresponding to the data in Fig. 4 in the main text, these values are estimated as $|X|^2 = 0.40, \gamma_0/\gamma_{pump} \approx 1$ for $k/k_0 = 0.024$ and $|X|^2 = 0.12, \gamma_0/\gamma_{pump} \approx 2$ for $k/k_0 = 0.078$. At the maximum fluence 3 μJ cm^{-2} this corresponds to polariton densities 3.1×10^4 μm^{-2} for $k/k_0 = 0.024$ and 4.5×10^4 μm^{-2} for $k/k_0 = 0.078$, yielding polariton–polariton interaction strength of $g_{\mathrm{P}}(k_x^{(2)}) \sim 0.160 \pm 0.014$ µeV·µm^2 and $g_{\mathrm{P}}(k_x^{(1)}) \sim 0.043 \pm 0.004$ µeV·µm^2, respectively. From the dependence of the polariton shift on the exciton Hopfield coefficient X, the exciton–exciton interaction strength can then be evaluated as $g_{\mathrm{X}} \sim 1.0\ \mu\mathrm{eV} \cdot \mu\mathrm{m}^2$. The statistical experimental uncertainty is estimated from fit errors, including spectral Fano fits, linear fits of blueshift vs. fluence, and fitting of the polariton-polariton interaction strength vs. Hopfield coefficient. For the extracted exciton–exciton interaction strength, this uncertainty amounts to $\sim 30\%$.

We note that the careful evaluation of the polariton density is very important for the calculation of the interaction strength. In order to eliminate potential sources of uncertainty

in the estimation of polariton density, we perform the nonlinear experiment under the following conditions: (i) polaritons are excited resonantly both in energy and wavevector, with spreads in E and k roughly corresponding to those of the polariton dispersion, minimizing the effect from other quasiparticles that might otherwise be created by the laser pulses, (ii) in real space, polaritons are excited uniformly with a large laser spot covering the entire $MoSe_2$ flake area, which ensures that the polaritons remain in the laser spot within the pulse duration, (iii) incident fluence is kept in the uJ/cm^2 range to avoid potential effects related to exciton-exciton annihilation. The remaining systematic uncertainty in our calculation of polariton density is due to possible deviation of the $\sim$ 200 µm laser spot shape on the sample from Gaussian and deviation of the actual laser pulse shape in time domain from the model. We estimate these contributions to be within 20%, giving the final estimate of the experimental exciton–exciton interaction strength of $g_{\mathrm{X}} = 1.0 \pm 0.4\ \mu\mathrm{eV} \cdot \mu\mathrm{m}^2$

SUPPLEMENTARY NOTE 6. EXCITON NONLINEARITY

We measure the pump-dependent blueshift of the exciton resonance in reflectance with TM-polarized excitation. The corresponding reflectance spectra are shown in the top panel of Fig. S9a for selected values of fluence varying from 0.1 µJ/cm^2 (top) to 1.8 µJ/cm^2 (bottom), with corresponding Lorentzian curves based on the extracted peak position and linewidth shown in the bottom panel. We fit the experimentally measured spectra with a Fano-like shape to obtain pump-dependent blueshift shown in the top panel of Fig. S9b with open squares. The exciton concentration for each fluence can be evaluated in the linear regime (omitting the effects of phase space filling) from the differential equation for the exciton annihilation operator b:

$$\frac{db}{dt} = -i\omega_X b - \frac{1}{2}(\gamma_0 + \gamma_X^h)b + \frac{\sqrt{\gamma_0}}{2}(1+r)a_0 \exp[-\frac{t^2}{2\tau_p^2}] \exp[-i\omega t], \tag{9}$$

where τ_p is the pulse duration, r is the reflection coefficient of the substrate, and a_0 is the square root of the number of photons passing through the monolayer per unit time per unit area, which is related to the peak incident power density W as $a_0 = \sqrt{W/(\hbar\omega)}$. We have substituted the bosonic operator with the complex amplitude. This differential equation can

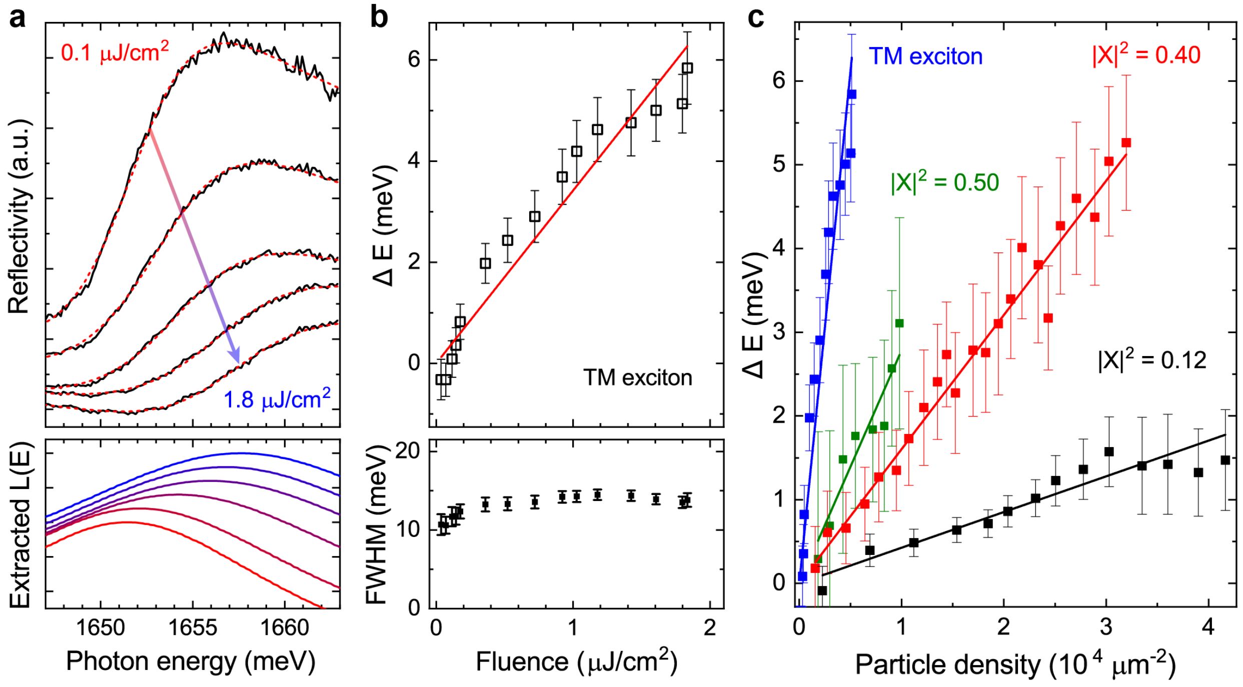

FIG. S9. Nonlinearity of excitons in TM polarization. (a) Top panel: measured exciton reflectance spectra (solid black curves) under resonant illumination with laser pulses for varying fluence, together with corresponding Fano fits (red dashed curves). The arrow indicates changing with power exciton resonance frequency. Bottom panel: corresponding Lorentzian curves based on the extracted peak position and linewidth. (b) Top panel: extracted excitonic spectral blueshifts as a function of incident laser fluence (symbols), together with corresponding linear fit (line). Bottom panel: corresponding extracted linewidth as fwhm as a function of fluence. (c) Nonlinear blueshifts of polaritons with indicated excitonic fractions (black, red, and green symbols) and exciton (blue symbols, probed in TM polarization) as functions of particle density, together with corresponding linear fits.

be solved explicitly yielding for $N = |b|^2$

$$N =$$
$$= |1+r|^2 \frac{|a_0|^2}{\gamma_0}(\gamma_0\tau_p)^2 \frac{\pi}{4} \left| \text{erf}\left[\frac{t}{\sqrt{2}\tau_p} + \frac{i\tau_p}{\sqrt{2}}(\delta + i\gamma/2) \right] + 1 \right|^2 \exp\left[-\tau_p^2(\delta^2 - \gamma^2/4) - \gamma t \right], \tag{10}$$

where $\delta = \omega - \omega_X$, $\gamma = \gamma_0 + \gamma_X^h$, and $t = 0$ is at the pulse centre. To account for the inhomogeneous broadening, we should convolve the expression for N with the gaussian distribution

of the exciton frequency with the width γ_X^{inh}:

$$\tilde{N} = \frac{1}{\sqrt{\pi}\gamma_X^{\mathrm{inh}}} \int d\delta N(\delta) e^{-\delta^2/\gamma_X^{\mathrm{inh}}} \tag{11}$$

This integral can not be taken analytically. For $\gamma_0 \approx \gamma_X^h = 1$ meV, $\tau_p = 130$ fs, and $\gamma_X^{\mathrm{inh}} = 8.4$ meV, the numerical integration returns a function of time with a maximum at $\xi_{max} \approx 0.8$.

In the case of TM polarization the reflection coefficient is almost constant across the pulse bandwidth, and $r \approx -0.25 - 0.35i$, yielding $|1+r|^2 = 0.68$. a_0 can be expressed through fluence F as $|a_0| = \sqrt{F/\tau_p/\sqrt{\pi}\hbar\omega_X}$.

Finally, the exciton concentration N for fluence F will be

$$\tilde{N}[\mu\mathrm{m}^{-2}] \approx 2800F[\mu\mathrm{J}\cdot\mathrm{cm}^{-2}]. \tag{12}$$

The measured exciton blueshift is plotted in Fig. S9c as a function of particle density (blue symbols). For comparison, we show power-dependent blueshifts measured for polaritons with different excitonic fractions ($|X|^2 = 0.12$, $|X|^2 = 0.40$, and $|X|^2 = 0.50$) as functions of polariton density (black, red, and green symbols). We evaluate g_{X} via linear fitting of the experimental data as $g_{\mathrm{X}} = 1.4 \pm 0.2\ \mu\mathrm{eV}\cdot\mu\mathrm{m}^2$.

We compare the obtained g_{X} value with a theoretical estimate [13] of $g_{\mathrm{X}} \sim 2E_{1s}r_{1s}^2 \sim 1.6\ \mu\mathrm{eV}\cdot\mu\mathrm{m}^2$, where $E_{1s} \sim 0.44$ eV is the $1s$ exciton binding energy in monolayer $MoSe_2$ and $r_{1s} \sim 1.36$ nm is the corresponding exciton Bohr radius [14].

---

* Corresponding author: vasily.kravtsov@metalab.ifmo.ru

† Corresponding author: ekaterina.khestanova@metalab.ifmo.ru

‡ Corresponding author: i.iorsh@metalab.ifmo.ru

[1] Grepstad, J. O. *et al.* Finite-size limitations on quality factor of guided resonance modes in 2D photonic crystals. *Opt. Express* **21**, 23640–23654 (2013).

[2] Nada, M. Y., Othman, M. A. & Capolino, F. Theory of coupled resonator optical waveguides exhibiting high-order exceptional points of degeneracy. *Phys. Rev. B* **96**, 184304 (2017).

[3] Sadrieva, Z. & Bogdanov, A. Effect of finite lateral size of dielectric grating on optical bound state in the continuum. In *J. Phys.: Conf. Ser.*, vol. 1092, 012127 (IOP Publishing, 2018).

[4] Li, L. New formulation of the Fourier modal method for crossed surface-relief gratings. *J. Opt. Soc. Am. A* **14**, 2758–2767 (1997).

[5] Houdré, R., Stanley, R. & Ilegems, M. Vacuum-field Rabi splitting in the presence of inhomogeneous broadening: Resolution of a homogeneous linewidth in an inhomogeneously broadened system. *Phys. Rev. A* **53**, 2711 (1996).

[6] Whittaker, D. *et al.* Motional narrowing in semiconductor microcavities. *Phys. Rev. Lett.* **77**, 4792 (1996).

[7] Mattuck, R. D. *A guide to Feynman diagrams in the many-body problem* (Courier Corporation, 1974).

[8] Ajayi, O. A. *et al.* Approaching the intrinsic photoluminescence linewidth in transition metal dichalcogenide monolayers. *2D Mater.* **4**, 031011 (2017).

[9] Scuri, G. *et al.* Large excitonic reflectivity of monolayer MoSe2 encapsulated in hexagonal boron nitride. *Phys. Rev. Lett.* **120**, 037402 (2018).

[10] Fang, H. *et al.* Control of the exciton radiative lifetime in van der Waals heterostructures. *arXiv preprint arXiv:1902.00670* (2019).

[11] Barachati, F. *et al.* Interacting polariton fluids in a monolayer of tungsten disulfide. *Nat. Nanotechnol.* **13**, 906 (2018).

[12] Fan, S., Suh, W. & Joannopoulos, J. D. Temporal coupled-mode theory for the Fano resonance in optical resonators. *J. Opt. Soc. Am. A* **20**, 569–572 (2003).

[13] Shahnazaryan, V., Iorsh, I., Shelykh, I. & Kyriienko, O. Exciton-exciton interaction in transition-metal dichalcogenide monolayers. *Phys. Rev. B* **96**, 115409 (2017).

[14] Li, J., Zhong, Y. & Zhang, D. Excitons in monolayer transition metal dichalcogenides. *J. Phys. Condens. Matter* **27**, 315301 (2015).
